# Epitope-based therapeutic targets in HCV genotype 1 non-structural proteins: a novel strategy to combat emerging drug resistance

**DOI:** 10.3389/fcimb.2024.1480987

**Published:** 2024-11-07

**Authors:** Mireayi Tudi, Adili Sawuti, Maimaitituerhong Abudurusuli, Chao Wu, Xiaoyu Chen, Gulimire Ailimu, Kuerbannisa Wulayin, Maimaitiaili Tuerxun

**Affiliations:** Department of Infectious Diseases, The First People’s Hospital of Kashi Prefecture, Kashi, China

**Keywords:** drug resistance, epitopes, hepatitis C, HLA allele, non-structural proteins

## Abstract

**Introduction:**

The hepatitis C virus (HCV) poses a major global health challenge, with its non-structural proteins being essential for viral replication and pathogenesis. Mutations in these proteins significantly contribute to drug resistance, necessitating innovative therapeutic strategies. This study aims to identify epitope-based therapeutic targets in the non-structural proteins of HCV genotype 1, employing in-depth in silico tools to counteract emerging drug resistance.

**Methods:**

We retrieved approximately 250 sequences of each non-structural protein from the NCBI database, capturing a broad spectrum of variability and sequence alignments, variability analysis and physicochemical property analysis were conducted. We utilized the TEPITOOL server by IEDB to predict cytotoxic T lymphocyte (CTL) epitopes. Following this, we assessed the efficiency of TAP transport and proteasomal cleavage using IEDB’s combined predictor tool. The epitopes were selected based on conservancy analysis, immunogenicity, allergenicity, and presence in non-glycosylated regions, ensuring high predictive scores and suitability as vaccine candidates. Epitopes were docked with the HLA-A*02:01 allele and Toll-like receptor-3 using the ClusPro server. The immune response potential of the epitopes was evaluated through *in-silico* immune stimulation.

**Results:**

The study identified 27 potential CTL epitopes from the non-structural proteins, including NS3, NS4a, NS4b, NS5a, and NS5b. Out of these, three lead epitopes demonstrated high conservation (>90%), strong binding affinities to HLA-A*02:01 and TLR-3, and robust immune response potential. These epitopes also showed favorable characteristics such as being non-allergenic and non-glycosylated.

**Conclusion:**

This comprehensive *in-silico* analysis provides a promising foundation for developing an epitope-based vaccine targeting HCV non-structural proteins, offering a novel approach to overcoming drug resistance in HCV treatment.

## 1 Introduction

Hepatitis C virus (HCV) is a leading cause of chronic liver disease, affecting approximately 58 million people worldwide, with 1.5 million new infections occurring annually (Kenfack-Momo et al., 2024). Chronic HCV infection can lead to severe liver conditions, including cirrhosis and hepatocellular carcinoma (HCC), significantly contributing to global morbidity and mortality with over 3,50,000 fatalities each year (Organization, W. H. 2019). Among the six genotypes of HCV (1 to 6), genotype 1 is the most prevalent and poses a considerable challenge due to its high propensity for developing resistance to antiviral therapies (Shen et al., 2023). The primary treatment for HCV involves direct-acting antivirals (DAAs), which target specific non-structural proteins of the virus, such as NS3/4A protease, NS5A, and NS5B polymerase (Dobrowolska et al., 2024). These DAAs have revolutionized HCV treatment, achieving high cure rates. However, the emergence of resistance-associated substitutions (RASs) in the HCV genome remains a significant obstacle, diminishing the efficacy of these drugs and necessitating the development of alternative therapeutic strategies (Xiong and Guo, 2024). Non-structural proteins of HCV, including NS3, NS4a, NS4b, NS5a, and NS5b, are crucial for the viral life cycle and are common targets for DAAs (Ramos et al., 2023). Mutations in these proteins can lead to resistance, underscoring the need for novel approaches to counteract this challenge. For instance, the NS3/4A protease is crucial for processing the HCV polyprotein, which is essential for viral replication. Similarly, NS5A plays a role in viral replication and assembly, while NS5B functions as an RNA-dependent RNA polymerase, which is vital for the replication of the viral genome (Izhari, 2023). The pivotal roles of these proteins in the HCV life cycle make them prime candidates for targeted therapeutic interventions. Epitope-based vaccines offer a promising approach to overcoming drug resistance in HCV. These vaccines are designed to elicit a robust and specific immune response against conserved regions of the virus, thereby enhancing the immune system’s ability to control and clear the virus, even in the presence of drug-resistant variants (Cowton et al., 2021). The strategy involves identifying short sequences of amino acids (epitopes) that are recognized by cytotoxic T lymphocytes (CTLs). CTLs play a critical role in controlling viral infections by targeting and destroying infected cells, making them a key component in the fight against HCV. The HLA-A*02:01 allele is one of the most prevalent human leukocyte antigen (HLA) class I molecules, known for its effective presentation of viral epitopes to CTLs (Wang et al., 2020). Predicting HLA-A*02:01 restricted epitopes from HCV non-structural proteins is, therefore, a strategic approach for developing potent vaccines. *In-silico* methods provide an efficient means of identifying potential epitopes, enabling the screening of extensive viral sequences and predicting epitopes with high binding affinity to HLA molecules (Sohail et al., 2021). The advancements in bioinformatics, have propelled vaccine science into a new era, introducing a contemporary, innovative, and highly practical approach to developing next-generation robust immunogens (Shawan et al., 2023). The methodology has previously been employed for developing vaccines against various pathogens like Influenza virus, Trypanosoma cruzi, Ebola and Dengue viruses and several others (Martinelli, 2022).

In this study, we aim to develop an epitope-based therapeutic strategy specifically targeting the non-structural proteins of HCV genotype-1 (Cowton et al., 2021). We hypothesize that by identifying HLA-A*02:01 restricted epitopes from NS3, NS4a, NS4b, NS5a, and NS5b proteins, we can formulate a vaccine that elicits a potent CTL response. This response would be capable of targeting and eliminating HCV-infected cells, thus providing a robust defense against the virus, even in the presence of drug-resistant mutations. By focusing on conserved regions within these non-structural proteins, we aim to create a vaccine candidate that not only prevents infection but may also offer therapeutic benefits. This includes enhancing the immune system’s ability to control and clear the virus, particularly in individuals infected with drug-resistant HCV variants. The outcomes of this research could pave the way for novel vaccine designs, contributing to the global effort to eradicate HCV and mitigate the impact of drug resistance.

## 2 Methodology

### 2.1 HCV non-structural proteins sequence retrieval and physicochemical properties analysis

The non-structural protein sequences for NS3, NS4a, NS4b, NS5a and NS5b of HCV genotype-1 were retrieved from NCBI database https://www.ncbi.nlm.nih.gov/. To capture a comprehensive view of the protein variations, we downloaded approximately 250 sequences from diverse regions, ensuring a broad representation of genetic variability. The inclusion Criteria for selecting the target sequences were: Genotype specificity: Sequences must belong to HCV genotype 1, as verified through BLAST analysis https://blast.ncbi.nlm.nih.gov/Blast.cgi; Protein specificity: Only sequences corresponding to the non-structural proteins NS3, NS4a, NS4b, NS5a, and NS5b were included; Sequence completeness: Full-length protein sequences were selected to ensure comprehensive analysis. Geographic diversity: Sequences were chosen to represent a broad range of geographic regions to capture genetic variability; and lastly Quality and annotation: Sequences must be well-annotated with minimal ambiguous bases, ensuring high-quality data. The exclusion criteria included Incomplete Sequences: Partial or truncated sequences were excluded to avoid incomplete data representation; Non-genotype 1 sequences: Any sequence not verified as belonging to HCV genotype 1 through BLAST analysis was excluded.; Low-quality sequences: Sequences with poor annotation, high levels of ambiguity, or those lacking sufficient metadata were excluded; Redundant sequences: Duplicates or highly similar sequences were excluded to avoid redundancy and ensure a diverse dataset; and Recombinant strains: Sequences identified as potential recombinants were excluded to maintain focus on standard genotype 1 variations. Initially, the sequences underwent BLAST analysis to confirm their identity and similarity. Subsequently, the aligned sequences were subjected to multiple sequence alignment using the COBALT tool (https://www.ncbi.nlm.nih.gov/tools/cobalt/re_cobalt.cgi) (Papadopoulos and Agarwala, 2007). This alignment was essential for identifying conserved and variable regions within the proteins. The physicochemical properties of the proteins like theoretical pI, mol. wt, extinction coefficient, estimated half-life, instability index, and grand average of hydropathicity (GRAVY) were then assessed using the PROTPARAM tool (https://web.expasy.org/protparam/) (Gasteiger et al., 2005).

### 2.2 Prediction of CTL epitopes using multiple algorithms

Initially, the amino acid sequences of each non-structural protein were analyzed using the TEPITOOL server (https://tools.iedb.org/tepitool/) by IEDB, one of the most advanced and up-to-date tools for epitope prediction (Paul et al., 2016). This tool enabled the identification of top-scoring epitopes based on their potential to elicit a CTL response. Following this, the top-scoring epitopes with percentile rank ≤ 1 were further evaluated for their efficient TAP transport and C-terminal proteasomal cleavage. This analysis was performed using the combined TAP transport and MHC class-I predictor tool provided by IEDB, which integrates these processes to predict the likelihood of an epitope being effectively presented on the surface of infected cells (Tenzer et al., 2005). TAP transport and proteasomal cleavage analysis are essential for evaluating the potential of epitopes as CTL targets. These processes are critical because proteasomal cleavage ensures that the epitope is processed and presented by the MHC, while TAP transport facilitates the transfer of these processed peptides into the endoplasmic reticulum for MHC loading. Both assessments are key to determining whether an epitope can be effectively recognized by CTLs.

### 2.3 Selection of promising epitopes

The initially identified epitopes were further refined based on several criteria to ensure their suitability for vaccine development.

### 2.4 Conservancy

Epitopes were examined for conservancy using the IEDB conservancy analysis tool (Bui et al., 2007). Epitopes demonstrating greater than 90% conservancy were selected for further analysis. Targeting conserved regions within the non-structural proteins of HCV genotype-1 is crucial because these regions are less likely to undergo mutations, which minimizes immune evasion and ensures the effectiveness of the vaccine against various viral strains, including drug-resistant ones.

### 2.5 Immunogenicity

The VaxiJen v2.0 server was utilized to evaluate the immunogenicity of the selected epitopes, effectively distinguishing between non-antigenic and antigenic epitopes (Doytchinova and Flower, 2007). Non-immunogenic epitopes were discarded. This was carried out in view that most of the peptides are less immunogenic, thus it becomes important to screen the epitopes for their immunogenicity.

### 2.6 Allergenicity

The remaining epitopes were evaluated for allergenicity using the AllerTOP v2.0 server (https://www.ddg-pharmfac.net/AllerTOP/method.html) (Dimitrov et al., 2014). Epitopes identified as allergenic were excluded.

### 2.7 Glycosylation

Epitopes were also checked for the presence of glycosylation sites using the NetNGlyc 1.0 server (https://services.healthtech.dtu.dk/services/NetNGlyc-1.0/) (Gupta and Brunak, 2001). Epitopes located in glycosylated regions were omitted to avoid potential issues with epitope processing and presentation.

The final list comprised epitopes that met all the criteria: high predictive scores, efficient TAP transport, proteasomal cleavage, conservancy, immunogenicity, non-allergenicity, and location in non-glycosylated regions. These promising epitopes were selected for further analysis.

### 2.8 Epitopes 3D structure and physicochemical analysis

The physicochemical properties of the epitopes as discussed in the methodology section were determined using the ProtParam server. The secondary structure of the epitopes was also analyzed. This analysis provided insights into the alpha helices, beta strands, and coils within the epitope structures, which are crucial for understanding their stability and potential interactions.

### 2.9 Evaluation of high-affinity CTL epitopes for HLA-A*02:01

The epitopes were further assessed for their affinity to the HLA-A*02:01 allele. To achieve this, the epitopes were modelled using the PEPFOLD 2.0 server (https://bioserv.rpbs.univ-paris-diderot.fr/services/PEP-FOLD/), which is a *de novo* tool for predicting the 3D structure of peptides between 9 and 36 amino acids in length (Shen et al., 2014). The secondary structural properties were also noted for all the epitopes as provided by the PEPFOLD server. PEPFOLD 2.0 uses Hidden Markov Models and performs simulations to generate the most representative conformations based on energy and population metrics. The predicted 3D structures of the epitopes were docked with the HLA-A*02:01 allele using the ClusPro server (Kozakov et al., 2017). ClusPro evaluates balanced docking energies, which include the combination of van der Waals interactions, electrostatics, and desolvation contributions, to predict the binding affinity of each epitope. The 3D model of HLA Class-I allele: HLA-A*02:01 was obtained from Protein Data Bank (PDB ID: 1QEW).

### 2.10 Optimization of epitopes through binding to the TLR-3 membrane receptor

To evaluate the immunogenic potential of the epitopes, their binding affinity to Toll-like receptor-3 (TLR-3) was further analyzed. The structure of the TLR-3 receptor was obtained from the Protein Data Bank (PDB ID: 7C76). The selected epitopes were docked with the TLR-3 receptor to evaluate their binding interactions. This step was crucial to ensure that the epitopes not only bind effectively to the HLA-A*02:01 allele but also engage with the TLR-3 receptor, which is vital for the innate immune response.

### 2.11 Immune simulation by lead epitopes

The immune response elicited by the lead epitope candidates, particularly the cytotoxic T-cell response, was assessed through *in-silico* immune simulations utilizing an ImmSim server (Rapin et al., 2010). The immune simulations were conducted using advanced *in-silico* tools with over 100 simulation steps. This comprehensive simulation aimed to model the immune response dynamics over time. The primary focus was on evaluating the cytotoxic T-cell response, which is crucial for targeting and eliminating HCV-infected cells. These immune simulations helped predict the potential effectiveness of the selected epitopes in generating a robust and targeted immune response, thereby validating their suitability as candidates for an epitope-based therapeutic strategy against HCV genotype-1.

## 3 Results

### 3.1 Target non-structural proteins

Approximately 250 protein sequences for each of the HCV non-structural proteins (NS3, NS4a, NS4b, NS5a, and NS5b) were retrieved in FASTA format from the NCBI database. These sequences represented a wide range of variability and geographical regions. The sequences were initially aligned using the protein BLAST tool from NCBI. The aligned sequences were then processed using the COBALT multiple sequence alignment tool. The COBALT alignment allowed us to visualize the sequences based on a frequency-based difference method. In this method, residues in a column are scored based on their representation in the column’s frequency profile. Rarely occurring residues are highlighted darkly, and columns with any degree of mismatch are also highlighted (**Supplementary Figure S1**). The physicochemical properties of these proteins were analyzed using the ProtParam tool. These analyses provided insights into various parameters such as molecular weight, theoretical pI etc, which are summarized in **Supplementary Table S1**. This comprehensive sequence alignment and physicochemical analysis laid the foundation for the subsequent identification and evaluation of potential epitopes.

### 3.2 HLA-A*02:01 targeting epitopes prediction

Epitopes targeting the HLA-A*02:01 allele were predicted using the TEPITOOL server, specifically focusing on 9-mer epitopes. The top-scoring epitopes were selected based on a consensus percentile rank of ≤1, ensuring high predictive accuracy. We employed the IEDB-recommended method for epitope prediction, which utilizes the default selection of predictors. This approach leverages the best available methods for a given MHC molecule, including artificial neural networks (ANN), stabilizing membrane models (SMM), and combinatorial libraries (CombLib) whenever applicable. Following the initial prediction, the selected epitopes were further evaluated for their likelihood of undergoing proteasomal cleavage and TAP transport. For this analysis, the sequences were evaluated using the Combined Predictor tool from IEDB, which integrates Proteasomal Cleavage, TAP Transport, and MHC Class I assessments. This comprehensive tool combines predictors for proteasomal processing, TAP transport, and MHC binding to deliver an overall score that indicates each peptide’s potential as a T cell epitope. This dual approach effectively led to the identification of potential epitopes characterized by high percentile ranks and favorable conditions for proteasomal cleavage, thereby enhancing their suitability as candidates for further development in an epitope-based therapeutic strategy (**Table 1**).

**Table 1:** Most promising epitopes predicted in each non-structural protein.

Non-structural protein	Epitope	Sequence	Conservancy	Vaxijen Score
NS3	E1	LVLNPSVAA	100%	0.5
	E2	VLNPSVAAT	100%	0.66
NS4a	E3	VLVGGVLAA	100%	0.43
	E4	AIIPDREVL	100%	0.58
NS4b	E5	GVAGALVAF	100%	0.48
	E6	RVTAILSSL	100%	0.49
NS5a	E7	GLGWAGWLL	83.6%	0.86
	E8	YVGGVEHRL	83.60%	1.16
	E9	ALSTGLLHL	100%	0.77
	E10	GLWSFSLLL	99.6%	0.6
	E11	SMMAFSAAL	83.6%	0.45
	E12	FVVSGLVGA	96.8%	0.8
	E13	GILSPGALV	83.6%	0.43
	E14	KVSARLLTL	97.2%	0.61
	E15	FQYSPAQRV	99.6%	0.43
	E16	GIVAPTMLV	97.2%	0.51
	E17	YLFNWAVKT	95.6%	0.68
	E18	LLLFVGVGL	88%	1.23
	E19	FVGVGLFLL	88%	0.95
NS5b	E20	SLLRHHNLV	100%	0.5
	E21	VTFDRLQVL	99.6%	0.53
	E22	VLDSHYQDV	98.4%	0.81
	E23	KVKANLLSV	100%	0.83
	E24	KAVAHINSV	97.6%	0.64
	E25	IMFAPTLWA	100%	0.71
	E26	YLFNWAVRT	100%	0.69
	E27	LLLAAGVGI	100%	0.83

E1-E27 are the epitope codes given along with their sequence.

### 3.3 Epitopes screening

The identified epitopes using the above-mentioned epitope prediction tools underwent a rigorous screening process based on several immunological parameters. Initially, the epitopes were assessed for conservancy, with only those exhibiting greater than 90% conservancy selected for further evaluation. The conserved epitopes were then subjected to immunogenicity testing using the VaxiJen v2.0. Non-immunogenic epitopes (below threshold 0.4) identified in this analysis were eliminated from consideration. The immunogenic epitopes were further subjected to allergenicity testing. The allergenic epitopes identified were also excluded from the final selection. Lastly, the epitopes were screened for their presence in glycosylation sites. Epitopes located in glycosylated regions were also eliminated from the list. As a result of this comprehensive screening process, we identified a set of epitopes that met the following criteria: high percentile scores, likelihood of undergoing proteasomal cleavage, high conservancy, immunogenicity, non-allergenicity, and non-glycosylation. The final predictions included two epitopes from NS3, two from NS4a, two from NS4b, thirteen from NS5a, and eight from NS5b (**Table 1**).

### 3.4 Epitopes affinity to HLA allele and TLR-3

**Figure 1:**
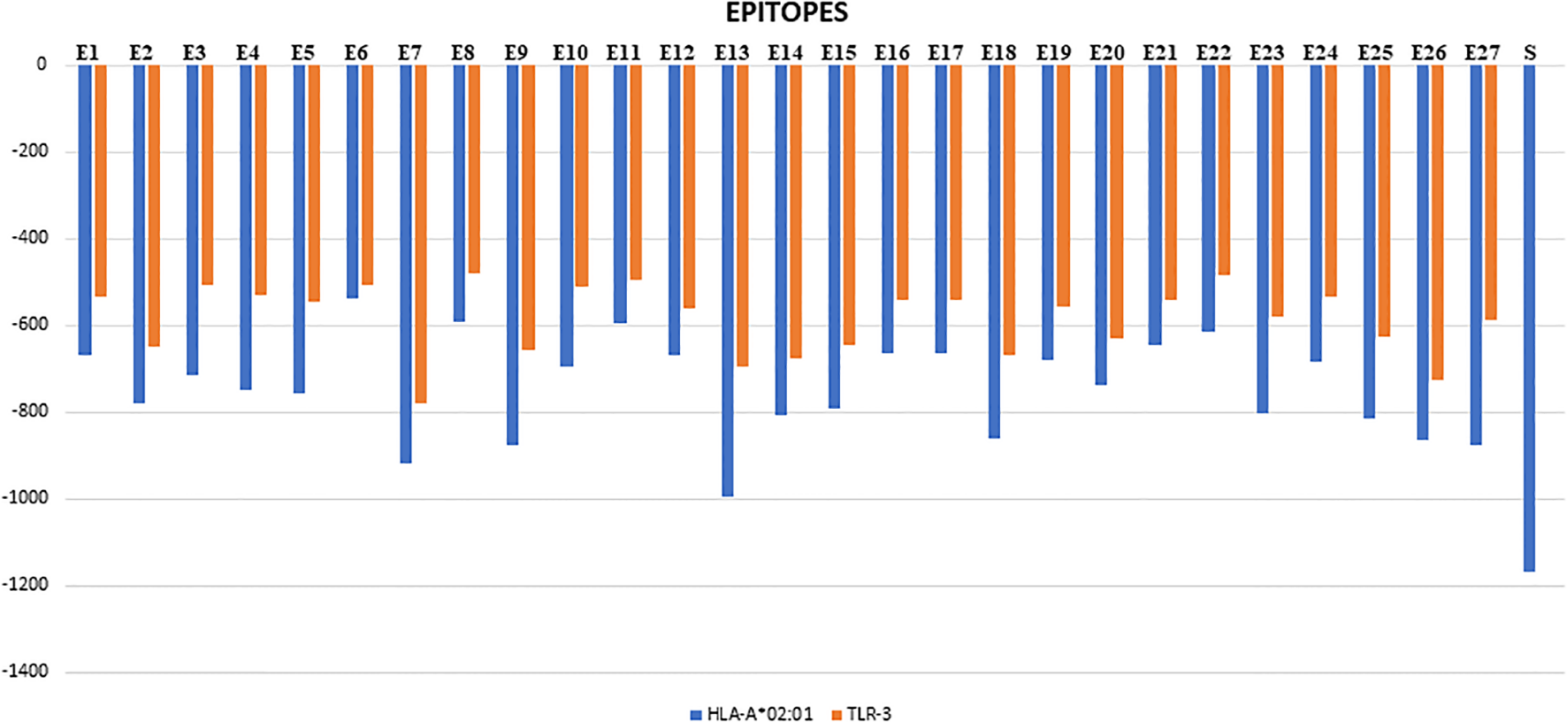
Bar graph representing the docking energies of epitopes (E1-E27) with HLA-A*02:01 allele and TLR-3 represented by blue and orange bars respectively. Std is the standard MAA-3 peptide.

**Figure 2:**
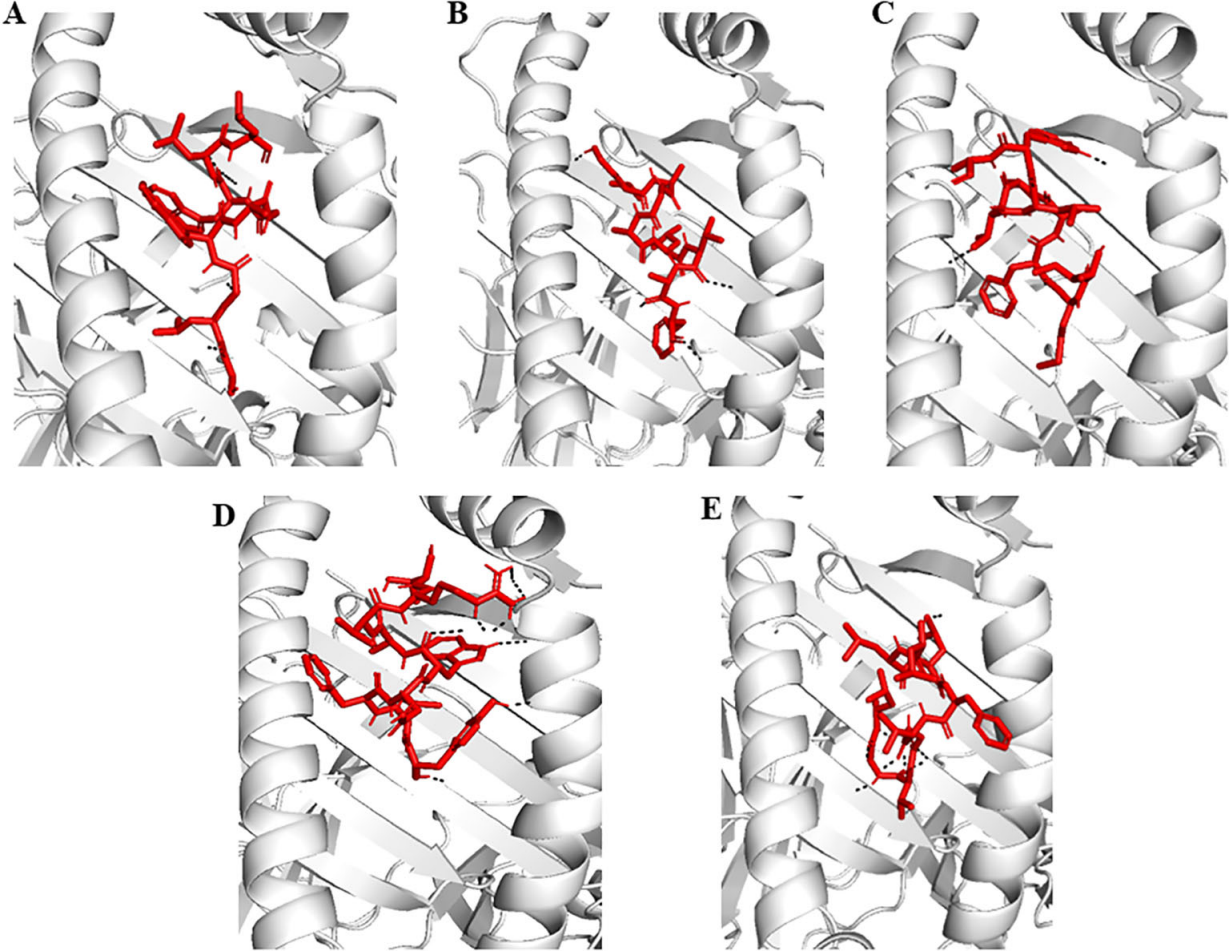
Representation of molecular docking pattern of epitopes with HLA-A*02:01 allele. The allele is represented in white (cartoon) and epitopes in red (sticks). The H-bonds formed are represented in black dashes. **(A–E)** are the epitopes E7, E9, E13, E14 and E27 respectively.

**Figure 3:**
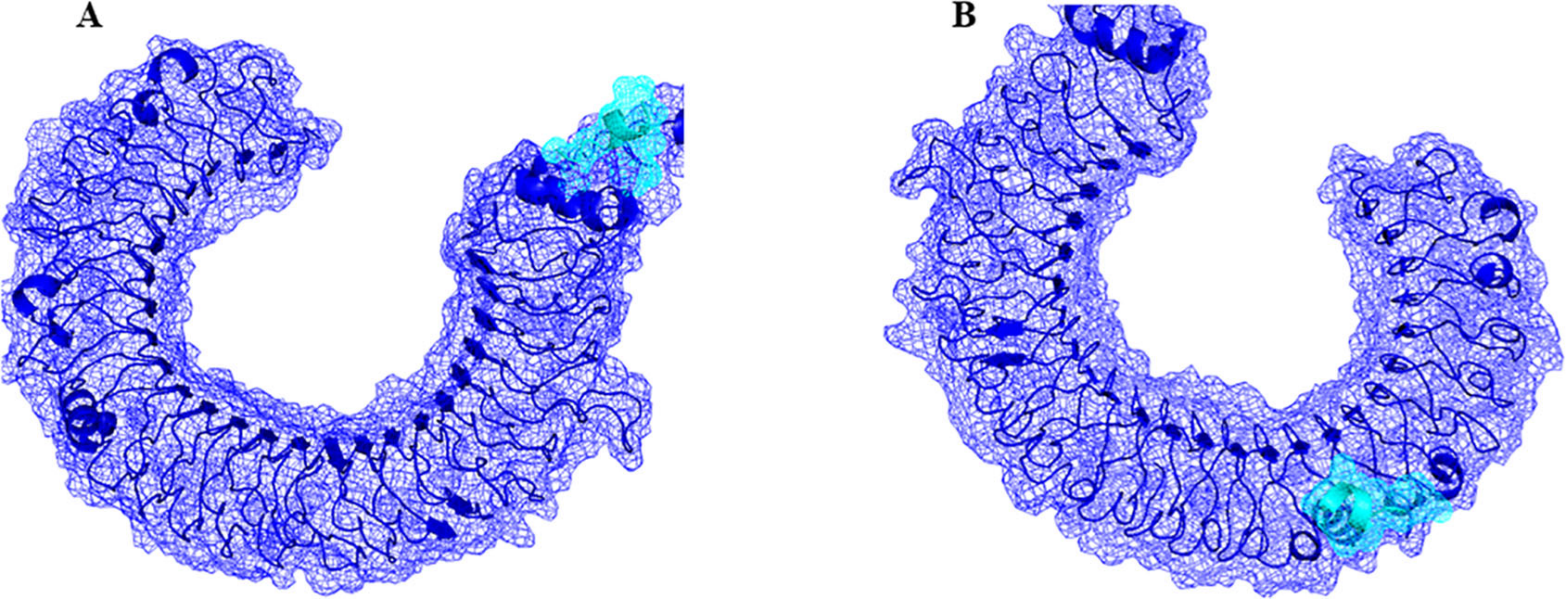
Cartoon representation of epitopes E7 **(A)** and E 13 **(B)** (cyan) with TLR-3 (dark blue).

**Figure 4:**
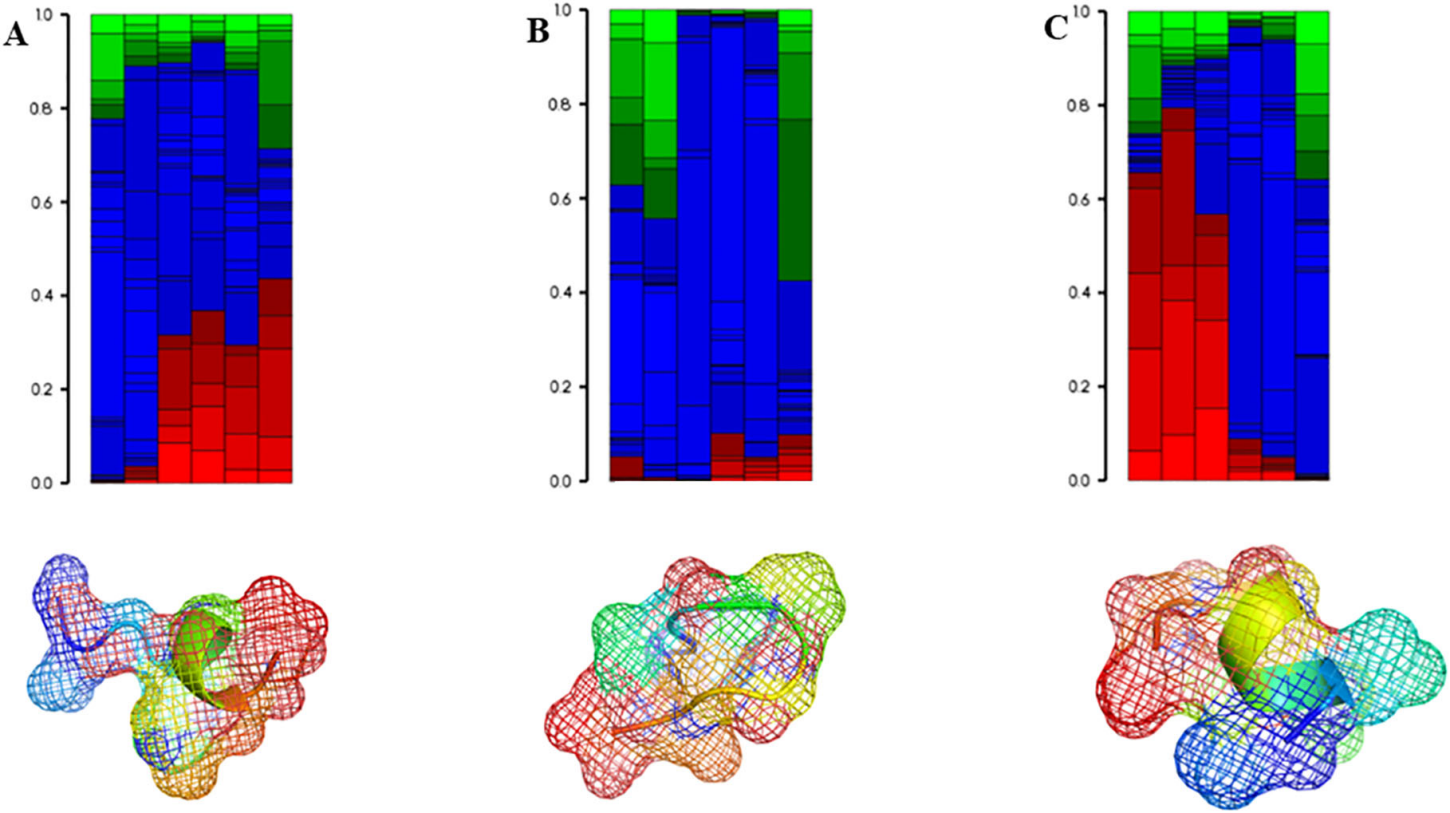
Secondary structural properties red: helical, green: extended and blue: coil of the top 3 lead epitopes. In addition, the 3D models of the epitopes are also represented in cartoons and mesh formats. **(A–C)** represent epitopes E7, E13 and E27.

The top-scoring epitopes were subjected to molecular docking analysis with the HLA-A02:01 allele using the ClusPro server (**Figure 1**). The 3D model of the HLA-A02:01 allele was extracted from the PDB, which includes a 9-mer melanoma-associated antigen-3 (MAA-3) complexed with the HLA-A*02:01 allele. Before docking, the retrieved 3D model underwent energy minimization and was prepared by removing the MAA-3 peptide, along with water molecules and other heteroatoms. All the epitopes were successfully docked in the active site of the HLA-A02:01 allele showing varying degrees of docking energy scores (**Figures 1**, **2**). In addition, the epitopes were also docked with TLR-3 in order to examine the affinities of epitopes towards immune receptors. The docked energy scores and docking patterns were examined and noted (**Figures 1**, **3**). Previously several studies have also utilized the ClusPro tool for studying molecular interactions between epitope and HLA molecules for vaccine development (Chauhan et al., 2021; Dubey et al., 2024). This analysis highlighted the potential of the epitopes to engage effectively with key immune components, supporting their candidacy for further development in an epitope-based therapeutic strategy. In addition, the secondary structural properties of the epitopes as provided by the Pepfold server were also examined (**Figure 4**).

### 3.5 Immune simulation analysis

**Figure 5:**
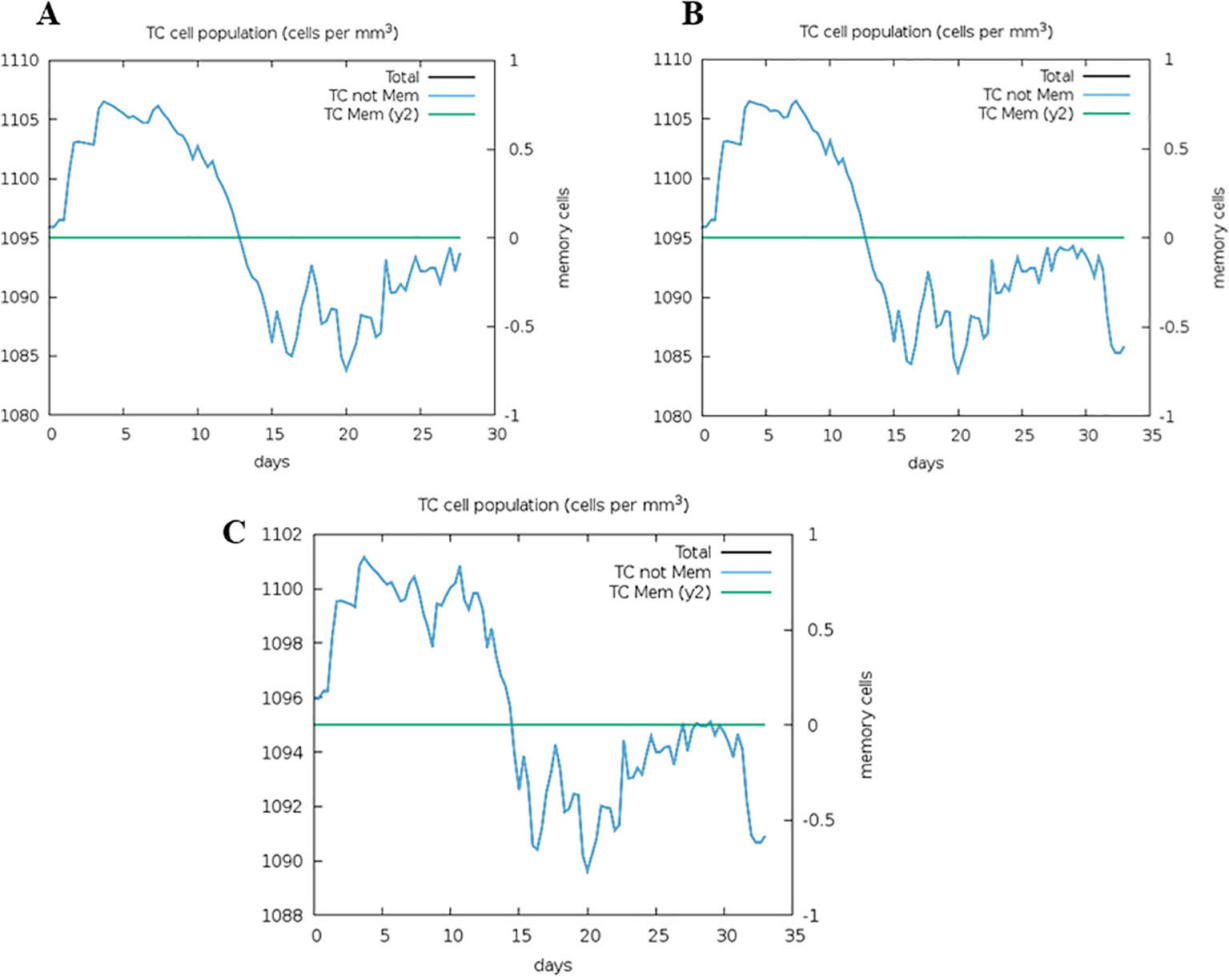
Immune simulation analysis of the epitopes E7 **(A)**, E13 **(B)** and E27 **(C)**. The capacity to induce the activation of cytotoxic T cells was investigated.

The top five epitopes with the highest affinities to the HLA-A*02:01 allele and TLR-3 i.e. epitopes E7, E9, E13, E14 and E27 underwent immune simulation analysis. Among these, epitopes E9, E13 and E27 demonstrated the strongest potential for activating cytotoxic T cells (**Figure 5**). This analysis facilitated the identification of lead epitopes that possess high affinities to both HLA and TLR, as well as key immunological characteristics: they are conserved, non-allergenic, immunogenic, non-glycosylated, and capable of inducing a robust cytotoxic T cell response.

## 4 Discussion

**Figure 6:**
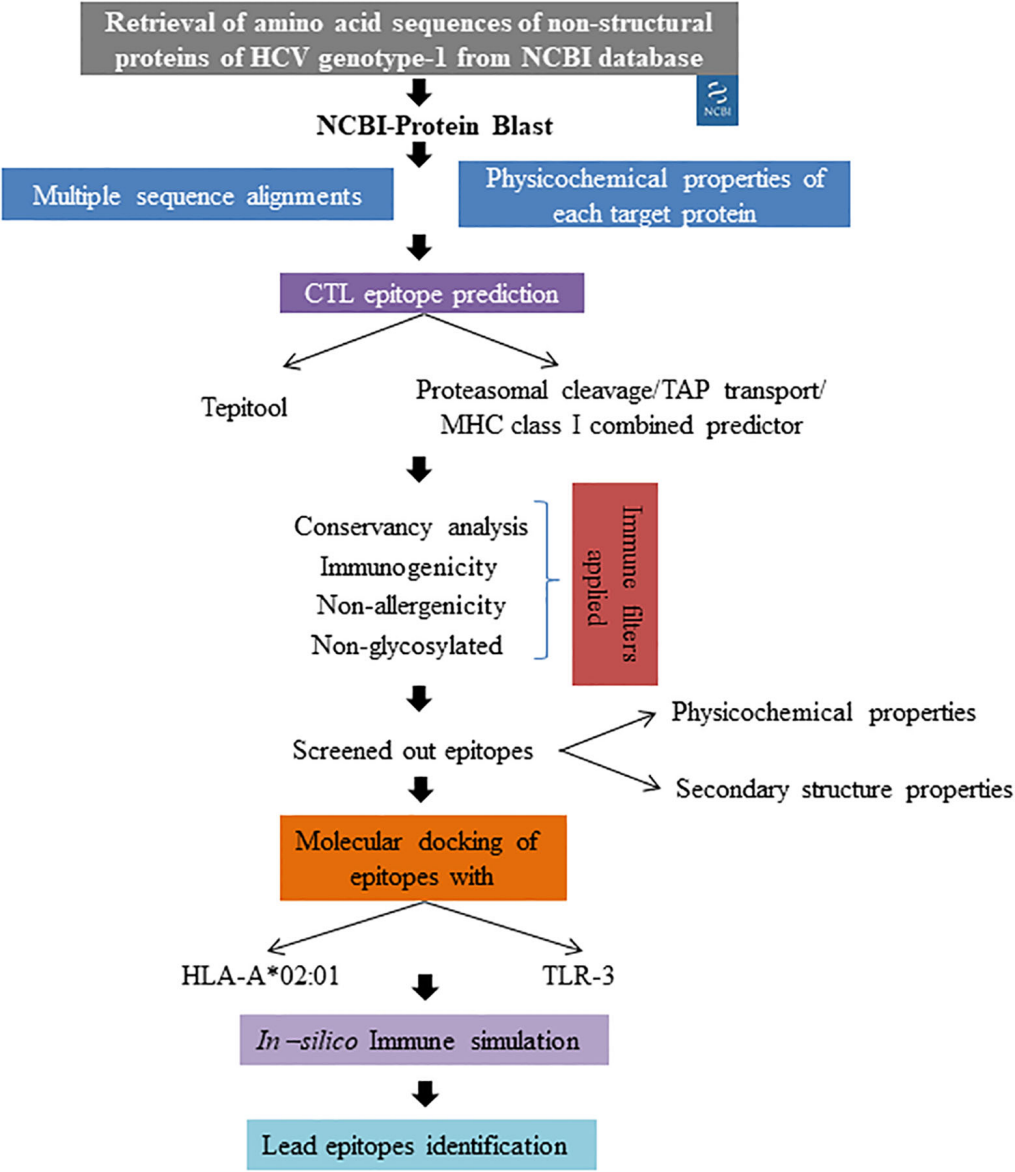
Overall methodology applied for lead epitope identification.

Hepatitis C virus (HCV) remains a significant global health challenge, primarily due to its ability to develop drug resistance, particularly in genotype-1, which is the most prevalent worldwide (Kenfack-Momo et al., 2024). Direct-acting antiviral (DAA) treatments achieve a cure rate of over 95% in individuals with chronic HCV infection. Nevertheless, for some patients where the treatment is unsuccessful, resistance-associated substitutions (RASs) may emerge, reducing retreatment options and increasing the potential for the transmission of resistant viruses (Howe et al., 2022). Our study aimed to develop an epitope-based therapeutic strategy specifically targeting the non-structural proteins of HCV genotype-1 to combat this rising resistance. The overall workflow employed for epitope screening is represented in **Figure 6**. Immuno-informatics is essential in vaccine development as it enables the identification of potential epitopes and the prediction of immune responses. This computational approach streamlines the design of effective vaccines against infectious diseases (Oli et al., 2020). Previous studies have successfully employed immuno-informatics for epitope prediction across various disease contexts, including cancers (Rizarullah et al., 2024), viral pathogens (Chauhan and Singh, 2020), and antimicrobial-resistant pathogens (Muteeb et al., 2024). By leveraging comprehensive immuno-informatics analyses, we identified 27 lead epitopes distributed across the targeted non-structural proteins: two from NS3, two from NS4a, two from NS4b, thirteen from NS5a, and eight from NS5b. Each of these epitopes exhibited high conservancy (>90%), non-allergenic properties, and robust immunogenicity, positioning them as promising candidates for vaccine development. Notably, our top five epitopes demonstrated significant binding affinities to both the HLA-A*02:01 allele and the TLR-3 receptor, further validating their potential as effective immunogens. The integration of multiple algorithms for predicting T cell epitopes has been shown to enhance predictive accuracy, evident in our selection process. This comprehensive methodology aligns with the practices highlighted in recent studies, such as Kumar et al (Kumar et al., 2021), who predicted CTL epitopes against SARS-CoV-2. Their approach involved multiple sequence alignments for conservancy analysis, the application of various epitope prediction algorithms, and a rigorous immune filtration process to screen effective T-cell epitopes. Furthermore, our findings resonate with those of Dobrowolska et al (Dobrowolska et al., 2024), who emphasized the importance of targeting conserved regions within viral proteins to elicit more effective and durable immune responses, especially in populations facing high rates of drug resistance. By focusing on conserved regions and employing a multi-faceted predictive strategy, our study aims to ensure that the identified epitopes can elicit strong and lasting immune responses. In comparison to earlier studies, such as that by Chauhan et al (Chauhan et al., 2018), which identified both B and T cell epitopes against Indian HCV genotype 3a using an immuno-informatics approach, our study stands out by utilizing a diverse range of global sequences. This broader dataset enhances the applicability and relevance of our findings across different populations and HCV strains. Moreover, our results suggest that the identified epitopes could form the foundation for a vaccine capable of not only preventing HCV infection but also inducing robust T cell-mediated responses against drug-resistant variants. We meticulously considered drug resistance mutations during the epitope screening process, ensuring that our selected epitopes remain relevant in the context of evolving viral strains. As highlighted by Izhari (2023), the continuously evolving nature of HCV necessitates ongoing monitoring of viral mutations and their effects on epitope presentation. Thus, continuous surveillance of circulating HCV strains will be vital in ensuring the long-term efficacy of any developed vaccine strategy. By integrating computational predictions with ongoing clinical and epidemiological studies, we can enhance our understanding of HCV dynamics and improve vaccine design strategies, ultimately contributing to more effective interventions against this persistent global health threat.

### 4.1 Limitations and future directions

While this study presents promising findings, the *in-silico* predictions must be validated through experimental and clinical studies. The identified epitopes should undergo further validation in preclinical settings, such as *in vitro* and *in vivo* immune response assays. Additionally, continuous monitoring of HCV mutations remains essential to ensure the ongoing relevance of the selected epitopes against emerging strains. Future research should focus on integrating these findings with population-based studies to determine the efficacy of these epitopes in diverse HCV-infected populations.

## 5 Conclusion

This study successfully identified lead epitopes targeting the non-structural proteins of HCV genotype-1, demonstrating high conservancy, non-allergenic properties, and robust immunogenicity. Our findings underscore the potential of these epitopes as candidates for vaccine development, particularly in combating drug-resistant variants. The top epitopes showed significant binding affinities to HLA-A*02:01 and TLR-3, validating their roles as effective immunogens. By employing a comprehensive immuno-informatics approach, we laid the groundwork for developing an epitope-based vaccine that could elicit strong cytotoxic T-cell responses. The reliance on computational predictions necessitates validation through experimental approaches, such as *in vitro* and *in vivo* studies, to confirm the immunogenic potential of the identified epitopes. Future research should focus on these experimental validations, as well as expanding the analysis to include a broader range of HCV genotypes and variants. Ongoing monitoring of HCV mutations will be essential to ensure the long-term efficacy of the identified epitopes and any future vaccine strategies. Ultimately, our research contributes to the advancement of effective therapeutic options against HCV infection and resistance.

## Data Availability

The datasets presented in this study can be found in online repositories. The names of the repository/repositories and accession number(s) can be found below: https://www.ncbi.nlm.nih.gov.
