# Supplementary material for: Epitope-based therapeutic targets in HCV genotype 1 non-structural proteins: a novel strategy to combat emerging drug resistance
Source: Front Cell Infect Microbiol. 2024 Nov 7;14:1480987. doi: 10.3389/fcimb.2024.1480987 (PMC11578958; doi:10.3389/fcimb.2024.1480987)
Supplement: Supplementary file 1 [file DataSheet1.docx]

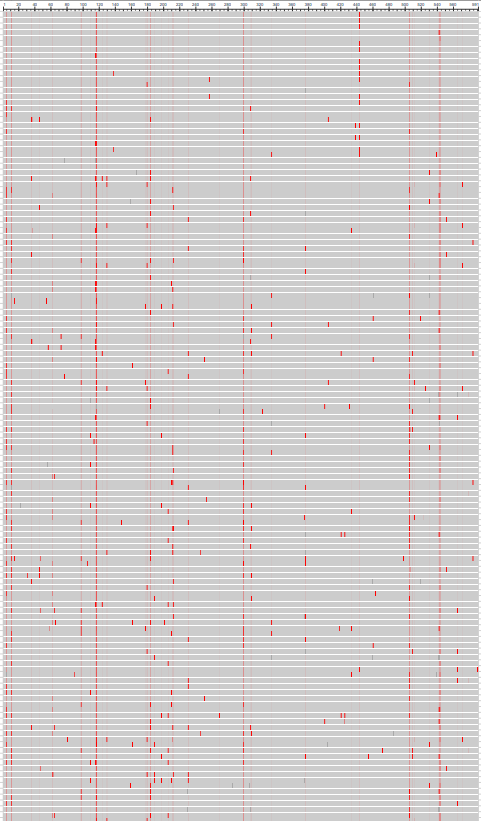

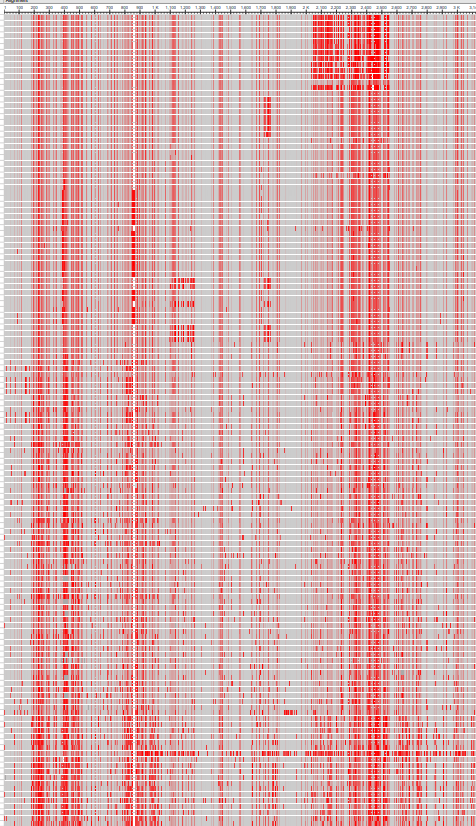

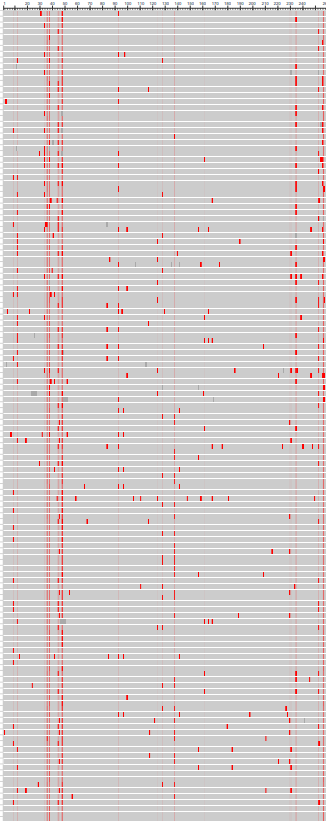

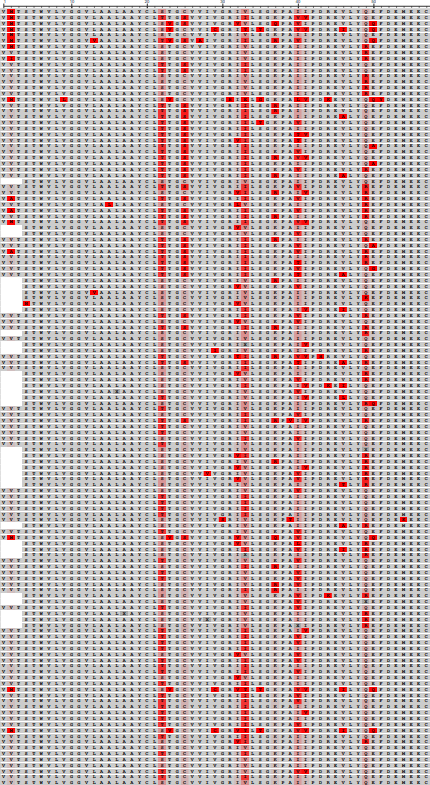

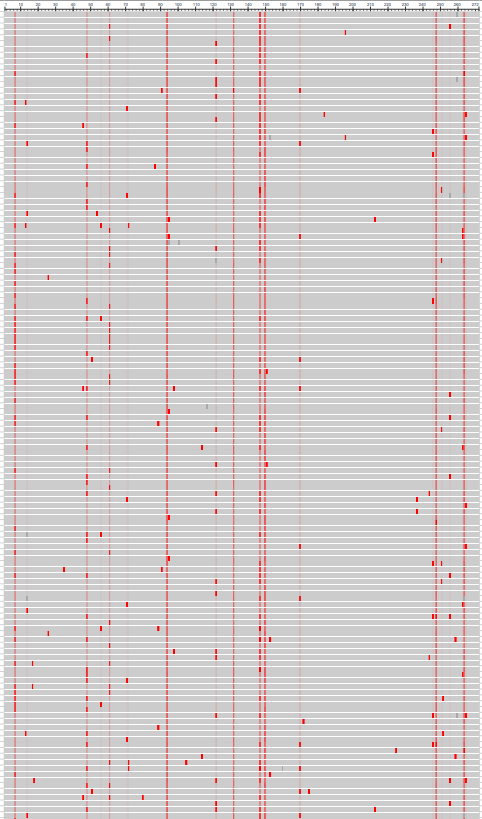


**E**

**D**

**A**

**B**

**C**

**Supplementary Figure 1:** Multiple sequence alignment of non-structural proteins of HCV genotype-1 using COBAL tool. A: NS3, B: NS4a, C: NS4b, D: NS5a, E: NS5b

| **Non-structural proteins** | **Mol. Wt.** | **T. pI** | **Ext.coef M^-1^ cm-^1^, at 280 nm** | **Estimated half-life (mammalian reticulocytes, in vitro)** | **Inst Index** | **Alip index** | **GRAVY** |
| --- | --- | --- | --- | --- | --- | --- | --- |
| NS3 | 28483.79 | 9.49 | 26275 | 4.4 hours | 37.46 | 79.56 | -0.010 |
| NS4a | 6686.88 | 4.49 | 8605 | 30 hours | 20.62 | 122.22 | -0.817 |
| NS4b | 27214.68 | 8.51 | 39085 | 30 hours | 42.56 | 109.23 | -0.504 |
| NS5a | 328194.08 | 8.66 | 531355 | 30 hours | 39.58 | 87.55 | -0.004 |
| NS5b | 65311.27 | 9.12 | 96145 | 1.9 hours | 41.41 | 86.70 | -0.103 |

Supplementary table 1: Physico-chemical properties of the non-structural proteins of HCV

.
